# Antibiotic resistance and biofilm formation among coagulase-negative staphylococci isolated from clinical samples at a tertiary care hospital of eastern Nepal

**DOI:** 10.1186/s13756-017-0251-7

**Published:** 2017-08-31

**Authors:** Lok Bahadur Shrestha, Narayan Raj Bhattarai, Basudha Khanal

**Affiliations:** Microbiology & Infectious Diseases, B. P. Koirala Institute of Health Sciences (BPKIHS), Dharan, Nepal

**Keywords:** CoNS, Nosocomial infection, Biofilm

## Abstract

**Background:**

Coagulase negative staphylococci were long regarded non-pathogenic as they are the commensals of human skin and mucosa but the recent changes in the medical practice and changes in underlying host populations, they are being considered significant pathogens associated with number of nosocomial infections. The objective of the study was to determine the species, antimicrobial susceptibility pattern, biofilm forming ability of the clinically significant CoNS isolates and to compare the different methods for the detection of biofilm formation.

**Methods:**

A total of 52 clinically significant CoNS isolates obtained from different units during a year period were studied. Characterization was done using standard microbiological guidelines and antimicrobial susceptibility was done following CLSI guidelines. Biofilm formation was detected by using three methods i.e. tissue culture plate method, congo red agar method and tube adherence method.

**Results:**

Among 52 isolates*, S. epidermidis* (52%) was the most common species which was followed by *S. saprophyticus* (18%) and *S. haemolyticus* (14%). Antimicrobial susceptibility pattern of CoNS documented resistance of 80% to ampicillin. Resistance to cefoxitin and ceftriaxone was observed in 58% of the isolates. Biofilm formation was observed in 65.38% of the isolates. The accuracy of Congo red agar and tube adherence method for the detection of biofilm formation was 82% and 76% respectively.

**Conclusion:**

CoNS isolates obtained from clinical samples should be processed routinely and antimicrobial susceptibility testing should be performed. Multidrug-resistant CoNS are prevalent. All the three methods i.e. tissue culture plate, Congo red agar and tube adherence method can be used in detecting biofilm formation.

## Background

Coagulase-negative staphylococci (CoNS) are the most abundant normal microbial flora of the human skin and mucous membranes. They were long regarded non-pathogenic as they are the commensals of human skin and mucosa. Due to the recent changes in the medical practice and changes in underlying host populations, they are being considered significant pathogens associated with a number of nosocomial infections [1, 2].

CoNS is now considered one of the most common causes of the device related infections in the recent two decades, especially in immunocompromised patients and the chronic debilitated patient who need the long-term central venous access [3]. They may adhere to medical devices and surfaces through slime layer which has a muco-polysaccharide structure, thus they may easily colonize and spread within the hospital environment. The slime factor also aids in pathogenicity by protecting them from phagocytosis, chemotaxis and also antimicrobial agents [4]. Studies suggest that selective advantage for CoNS in causing device associated infections is achieved by its ability to adhere to plastic catheters more aggressively than any other organism [5].

CoNS isolated from nosocomial environments are usually resistant to multiple antimicrobial agents. About 80%–90% of CoNS isolates associated with hospital infections are methicillin resistant coagulase-negative staphylococci (MRCoNS) [4].

With the advancement of medical sciences, especially with the increasing use of medical devices, the infections caused by CoNS are ever increasing. The characterization of CoNS upto species level, their antimicrobial properties and biofilm production is very important for its diagnosis and treatment. The present study is therefore proposed to determine the species distribution, antimicrobial susceptibility and surface adherence property of CoNS isolated at BPKIHS hospital.

## Methods

### Isolates

The study was carried out in the Department of Microbiology, B.P. Koirala Institute of Health Sciences, Dharan, Nepal from July 2013 to June 2014. Consecutive sampling method was used i.e. all the clinically significant CoNS isolates obtained during the study period were enrolled. A total of 52 CoNS isolates were obtained from various clinical specimens (blood, pus, urine, CVC, tracheostomy tube, tissue) were included in the study.

### Identification and characterization of CoNS

Bacterial isolates obtained were identified by colony morphology, gram staining and biochemical tests (catalase test, slide coagulase, tube coagulase and mannitol test). Once the identity of the isolate as CoNS was confirmed, several biochemical tests and antimicrobial discs were used for their characterization upto species level. All the tests were performed according to standard microbiological methodology [6, 7].

### Antimicrobial susceptibility test: [8, 9]

Antimicrobial susceptibility testing of the isolates was performed on Mueller-Hinton Agar by.

Kirby-Bauer disk diffusion method recommended by clinical laboratory standard institution.

(CLSI) guidelines against following antimicrobial discs (HiMedia Laboratories): amikacin, ampicillin, ceftriaxone, ciprofloxacin, azithromycin, cotrimoxazole, vancomycin and linezolid.

Cefoxitin disc was used to detect methicillin resistance. *Staphylococcus aureus* ATCC 25923 and *Escherichia coli* ATCC 25922 were used as control strains and tested along with the test strains.

### Determination of minimum inhibitory concentration (MIC) [10]

MIC of oxacillin and Vancomycin was determined by agar dilution technique as per CLSI guidelines. Oxacillin powder (815 μg/g) and Vancomycin powder (potency = 930 μg/g) was obtained from HiMedia laboratories India. The concentration at which complete inhibition of growth is achieved is considered the MIC. The results were interpreted according to the CLSI guidelines.

### Study of biofilm formation

Biofilm production were investigated by the tube adherence test described by Christensen et al. (1982) and Congo red agar method as described by Freeman et al. (1989) and Tissue culture plate method as described by Christensen et al. (1985) [5, 11, 12]. Results were interpreted considering Tissue culture plate method as gold standard [13–15].

### Tube adherence method [5]

A loopful of bacterial suspension from overnight culture were inoculated into the Trypticase.

Soy Broth with 1% glucose (TSBGlu, 10 mL) and incubated for 24 h at 37 °C. The tubes were gradually poured down and was washed with phosphate buffer solution (pH 7.3). After drying, the tubes were stained with 0.1% crystal violet. Excess stains were removed and tubes were washed away with water multiple times. Tubes were then kept in inverted position and finally observed for biofilm formation.

### Congo red agar method [11]

Congo red agar was prepared by mixing 37 g Brain heart infusion broth (HiMedia), 50 g sucrose (HiMedia), 0.8 g congo red dye and 10 g agar in 1 l distilled water. The solution was then sterilized by autoclave (121 °C, 15 lbs. for 15 min) and dispensed onto 90 mm petri dishes. CoNS isolates were plated and were incubated at 37 °C for 24 h. Biofilm production was detected by culturing the CoNS isolates on congo red agar plates. The biofilm forming strains produced black colonies while non-forming strains developed red colonies.

### Tissue culture plate (TCP) method [12]

Trypticase soy broth with 1% glucose (TSBglu) was used as culture media. Isolates from fresh agar plates were inoculated in TSBglu media and incubated for 18–24 h at 37 °C in aerobic condition. After incubation, 0.2 ml of culture was filled onto individual wells of 96 well flat-bottom tissue culture plates. For control to check sterility and non-specific binding of media, only broth without the bacteria was used. The tissue culture plates were incubated aerobically at 37 °C for 18–25 h. The plates were gently tapped for the removal of unbound content. For the removal of free floating bacteria, the individual wells were cleaned four times with 0.2 ml of phosphate buffer saline (PBS pH 7.2). The biofilms formed by adherent organisms in tissue culture plate were fixed with sodium acetate (2%) and stained with 0.1% crystal violet. The plates were washed with water for excess stain and were left for drying. The isolates which formed biofilm were adherent on all side wells and stained uniformly with crystal violet.

## Results

**Fig. 1:**
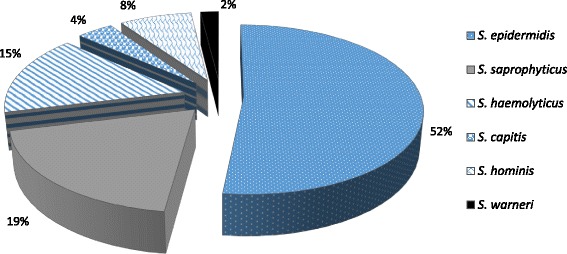
Different species of coagulase-negative staphylococci (*n* = 52)

A total of 52 CoNS isolates were studied over the duration of 1 year (July 2013–june 2014). The species identification was done on the basis of standard guidelines [6, 7] using the biochemical tests which is interpreted in Table 1. Among them, *S. epidermidis* (52%) was the most common species, followed by *S. saprophyticus* (18%) and *S. haemolyticus* (14%). The overall species distribution is further elicitated on Fig. 1.

**Table 1:** Biochemical tests for the identification of CoNS isolates and their interpretation

Biochemicals															Identification
Urease	Acetoin production	Novobiocin	Polymyxin B	Slide coagulase	Tube coagulase	Alkaline phosphatase	Pyrrolidonyl aryiamidase	Ornithine Decaboxylase	Trehalose	Mannitol	Mannose	Xylose	Maltose	Sucrose	
+	+	S	R	−	−	+	−	−	−	−	+	−	+	+	*S. epidermidis* (*n* = 27)
+	+	R	S	−	−	−	−	−	+	−	−	−	+	+	*S. saprophyticus* (*n* = 10)
−	+	S	S	−	−	−	+	−	+	−	−	−	+	+	*S. haemolyticus(n* = 8)
+	−	S	S	−	−	−	−	−	−	−	−	−	+	+	*S. hominis (n = 4)*
−	−	S	S	−	−	−	−	−	−	+	+	−	−	+	*S. capitis* (*n* = 2)
+	+	S	S	−	−	−	−	−	+	−	−	−	+	+	*S. warneri* (*n* = 1)

[+ = Positive, ─ = negative, R = Resistant, S = Sensitive]

**Fig. 2:**
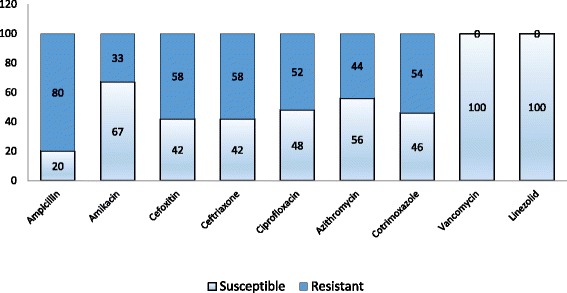
Antimicrobial susceptibility pattern of different CoNS isolates against several antimicrobial agents (%)

Antimicrobial susceptibility pattern of CoNS documented resistance of 80% of isolates to ampicillin. Resistance to ceftriaxone was observed in 58% of the isolates. Among 52 isolates,

Twenty two were susceptible to methicillin as indicated by cefoxitin disc diffusion (≤24 mm) and 30 were resistant. Methicillin resistance was confirmed by MIC of oxacillin against these isolates. All the isolates were susceptible to vancomycin and linezolid. Susceptibility to vancomycin was confirmed by calculating MIC of vancomycin against these isolates (Fig. 2).

The present study also showed that MRCoNS are comparatively more resistant to multiple antimicrobial agents. Isolates obtained from critical care units were more resistant strains as compared to isolates obtained from the outpatient department and wards.

### Study of biofilm formation

Biofilm formation was studied in this study by three methods i.e. Tissue culture plate method,

Congo red agar method and tube adherence method. Among 52 isolates, 34 isolates were biofilm formers; 34 of them were detected by tissue culture plate method, 29 by congo red agar method while 27 isolates were biofilm positive by tube adherence method.

The tissue culture plate method was used as the standard method to evaluate the congo red agar and tube adherence method [13–15]. Sensitivity, specificity, positive predictive value, negative predictive value, and accuracy were calculated by analysis of 2*2 table (Table 2). The sensitivity of congo red agar was 82.35% which is higher than tube adherence method 76.47%. The specificity of two tests was similar 94%. The accuracy of congo red agar method (86.5%) was also higher than tube adherence method (82.69%) (Table 3).

**Table 2:** Biofilm formation by Congo red agar, tube adherence and tissue culture plate methods

		Tissue culture plate method	
		Positive	Negative
Congo red method	Positive	28	1
	Negative	6	17
Tube adherence method	Positive	26	1
	Negative	8	17

**Table 3:** Statistical evaluation of Congo red agar and Tube adherence method for detection of biofilm lm formation in staphylococci using the tissue culture plate method as the standard method

	Sensitivity	Specificity	PPV	NPV	Accuracy
Congo red agar	82.35	94.5	96.5	43.47	86.5
Tube adherence method	76.47	94.44	96.29	68	82.69

Biofilm formation was seen most commonly in *S. epidermidis* followed by *S. haemolyticus.*

**Fig. 3:**
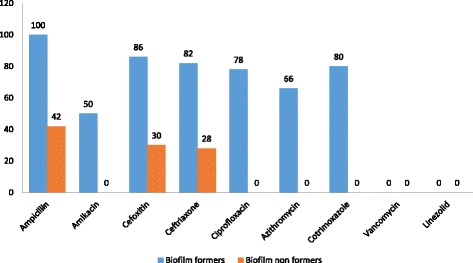
Antimicrobial resistance pattern with relation to biofilm formation property

The biofilm producers were resistant to multiple antibiotics in comparison to biofilm non- formers. Resistance to ampicillin and methicillin was seen in 100% and 86% of biofilm producers while only 42% and 30% resistance was seen in biofilm non-producers (Fig. 3).

The biofilm producer isolates were studied on the basis of the origin of the sample. All the isolates obtained from the intensive care unit (ICU) were biofilm former while no isolates obtained from outpatient department produced biofilm.

## Discussion

### General distribution and speciation

A total of 52 clinically significant isolates of coagulase-negative staphylococci obtained during the period of 1 year (July 2013–June 2014) were included in our study. Among the isolates, 6 different species of coagulase-negative staphylococci were encountered: *S. epidermidis* (27, 52%), *S. saprophyticus* (10, 19%), *S. haemolyticus* (8, 15%), *S. hominis* (4, 8%), S. capitis (2, 4%) and *S. warneri* (1, 2%). The findings of the present study are in agreement with the various studies which shows *S. epidermidis* as the most common coagulase-negative staphylococci [16, 17]. In contrast to the finding of our study, Jain A et al. isolated *S. haemolyticus* as (58%) as the most common isolate, followed by *S. epidermidis* (17%) [18]. *S. epidermidis* was isolated from various clinical specimens like blood, central venous catheter, pus and urine, while *S. saprophyticus* was predominantly obtained from urine samples (90%). The result correlates with the other studies which reported the pathogenic role of *S. epidermidis* in catheter related sepsis and that of *S. saprophyticus* in UTI [4, 5]. The higher incidence of *S. saprophyticus* in UTI is due to its colonization of the rectum or urogenital tracts of 5–10% of women [4].

### Antimicrobial resistance

In our study, the antimicrobial susceptibility of isolates was tested against 9 antimicrobials in accordance with the CLSI guidelines [8]. AST pattern of the isolates showed 80% resistance to ampicillin, 55% to ceftriaxone. Fifty-eight percent of the isolates were characterized as methicillin-resistant CoNS. All the 52 isolates were susceptible to vancomycin and linezolid. The susceptibility of vancomycin was confirmed by MIC against the isolates. The result of our study is consistent with other research article [19]. However some studies have suggested that vancomycin resistance does exist and is in increasing trends. Natoli S et al. observed no resistance to vancomycin but 5.4% isolates had reduced susceptibility to vancomycin [20].

Isolates obtained from intensive care unit (ICU + NICU) were more resistant when compared to other wards and outpatient department. A similar pattern of drug resistance was documented by several other studies [19, 21]. The patients hospitalized in intensive care unit are managed with multiple indwelling medical devices for the medication, nutrition and respiration. They are more vulnerable to biofilm formation and subsequent infection [22].

### Biofilm formation

Among the 52 isolates, 34 (65.38%) isolates were biofilm formers. Biofilm formation was detected by tissue culture plate method in 34 isolates, congo red agar in 29 isolates, while 27 isolates showed positive biofilm formation test by tube adherence method. Similar results were obtained in previous studies [23, 24].

Congo red agar detected 85% (29/34) biofilm producer and tube adherence method detected 79% (27/34). Tissue culture plate is used as the standard method for detection of biofilm formation [13–15] and the results of our study supports the evidence. Similar results were documented in various studies [13, 17].

The sensitivity, specificity, accuracy of the two tests were calculated using tissue culture plate as the reference [13–15]. The sensitivity (82.35%) and accuracy (86.5%) of congo red agar were higher than tube adherence method (sensitivity = 76.47, accuracy = 82.69%), while no difference was observed in the specificity of these two methods. The positive predictive value of both congo red agar and tube adherence method is good (above 90), and they may therefore be used in clinical decision making. Similar results were documented by Saising et al. [24].

The present study also reveals that 100% of isolates obtained from intensive care units were biofilm formers while only 40% of isolates from surgery ward and none of the isolates obtained from the outpatient departments were biofilm formers. Similarly, the biofilm producers were more resistant to antimicrobial agents as compared to non-producers which agree with the study done in the past [5].

## Conclusion

A total of 52 isolates of CoNS were studied, among which 6 species were identified. *S. epidermidis* (52%) was the most common species followed by *S saprophyticus* (19%), S. haemolyticus (15%) and *S. hominis* (8%). Antimicrobial susceptibility pattern of CoNS documented resistance of 80%, 58% and 58% resistance to ampicillin, ceftriaxone and cefoxitin respectively. All the isolates were susceptible to vancomycin and linezolid. Biofilm production was detected in 67% of the isolates. Tissue culture plate is the most effective method for the detection of biofilm formation while congo red agar and tube adherence method can also detect biofilm formation with an accuracy of 86% and 82% respectively.

## Abbreviations

AMR: Antimicrobial resistance
ATCC: American type culture collection
CLSI: Clinical and laboratory standards institute
CoNS: Coagulase-negative Staphylococci
CVC: Central vascular catheter
FDA: Food and Drug Administration
HIV: Human immunodeficiency virus
ICU: Intensive Care Unit
MIC: Minimum Inhibitory Concentration
MRCoNS: Methicillin-resistant Coagulse-negative Staphylococci
MSCoNS: Methicillin-sensitive Coagulase-negative Staphylococci
NICU: Neonatal intensive care unit
UTI: Urinary tract infection
